# Benign Hepatocellular Tumors in Children: Focal Nodular Hyperplasia and Hepatocellular Adenoma

**DOI:** 10.1155/2013/215064

**Published:** 2013-03-11

**Authors:** Stéphanie Franchi-Abella, Sophie Branchereau

**Affiliations:** ^1^Department of Pediatric Radiology, Hôpital Bicêtre, Hôpitaux Universitaires Paris-Sud, Assistance Publique Hôpitaux de Paris, 78 Rue du General Leclerc, 94278 Le Kremlin-Bicêtre, France; ^2^Department of Pediatric Surgery, Hôpital Bicêtre, Hôpitaux Universitaires Paris-Sud, Assistance Publique Hôpitaux de Paris, 78 Rue du General Leclerc, 94278 Le Kremlin-Bicêtre, France

## Abstract

Benign liver tumors are very rare in children. Most focal nodular hyperplasia (FNH) remain sporadic, but predisposing factors exist, as follows: long-term cancer survivor (with an increasing incidence), portal deprivation in congenital or surgical portosystemic shunt. The aspect is atypical on imaging in two-thirds of cases. Biopsy of the tumor and the nontumoral liver is then required. Surgical resection will be discussed in the case of large tumors with or without symptoms. In the case of associated vascular disorder with portal deprivation, restoration of the portal flow will be discussed in the hope of seeing the involution of FNH. HepatoCellular Adenoma (HCA) is frequently associated with predisposing factors such as GSD type I and III, Fanconi anemia especially if androgen therapy is administered, CPSS, and SPSS. Adenomatosis has been reported in germline mutation of HNF1-**α**. Management will depend on the presence of a predisposing factor and may include metabolic control, androgen therapy withdrawn, or closure of the shunt when appropriate. Surgery is usually performed on large lesions. In the case of adenomatosis or multiple lesions, surgery will be adapted. Close followup is required in all cases.

## 1. Introduction

Liver tumors are very rare in children, accounting for 1 to 4% of all pediatric tumors. Benign tumors account for 30 to 40% of these, with a majority of hemangiomas occurring during infancy. Focal nodular hyperplasia (FNH) and hepatocellular adenoma (HCAs) are extremely rare during childhood, and there are few published reported cases and series. Presentation, physiopathology, and management differ from adults.

We will successively review the main characteristics of FNH and HCA in children and discuss physiopathology, followup, and therapeutic modalities based on a systematic review of the literature and our experience. 

## 2. Focal Nodular Hyperplasia

### 2.1. Histological Definition and Physiopathology

Focal nodular hyperplasia (FNH) is not a neoplasm but a nonspecific hyperplasic reaction to vascular abnormalities. It is a well-delimitated lesion without capsules and characterized by hepatocytic nodules separated by fibrous bands. The mass has a central stellate fibrous region containing malformed vascular structures that include large arteries, without portal veins. Bile ductular reaction is usually present at the interface between hepatocytes and fibrous bands and is highly suggestive of the diagnosis of FNH. According to some authors, in FNH, arterial blood flows from the anomalous arteries via capillaries into sinusoids adjacent to the fibrous septa. The blood in the sinusoids drains to the hepatic vein either directly or via perinodular veins. The absence of portal vein branches in FNH leads to the absence of portal blood flow.

The precise cause of FNH is unknown. Several theories have been suggested to explain the occurrence of FNH: vascularization by an anomalous large artery, acquired thrombosis, reactive hyperplasia after hepatocellular injury induced by vasculitis, or higher blood flow, either portal or arterial, compared with the surrounding tissues [1–8].

### 2.2. Frequency and Predisposing Factors in the Pediatric Population

FNH is very uncommon in children. About 200 cases have been reported in the literature, with few short series [9–14]. It represents from 2% to 7% of pediatric liver tumors [10, 13, 15]. FNH has been reported in all pediatric age groups, including prenatal and neonatal forms [16–18].

**Figure 1:**
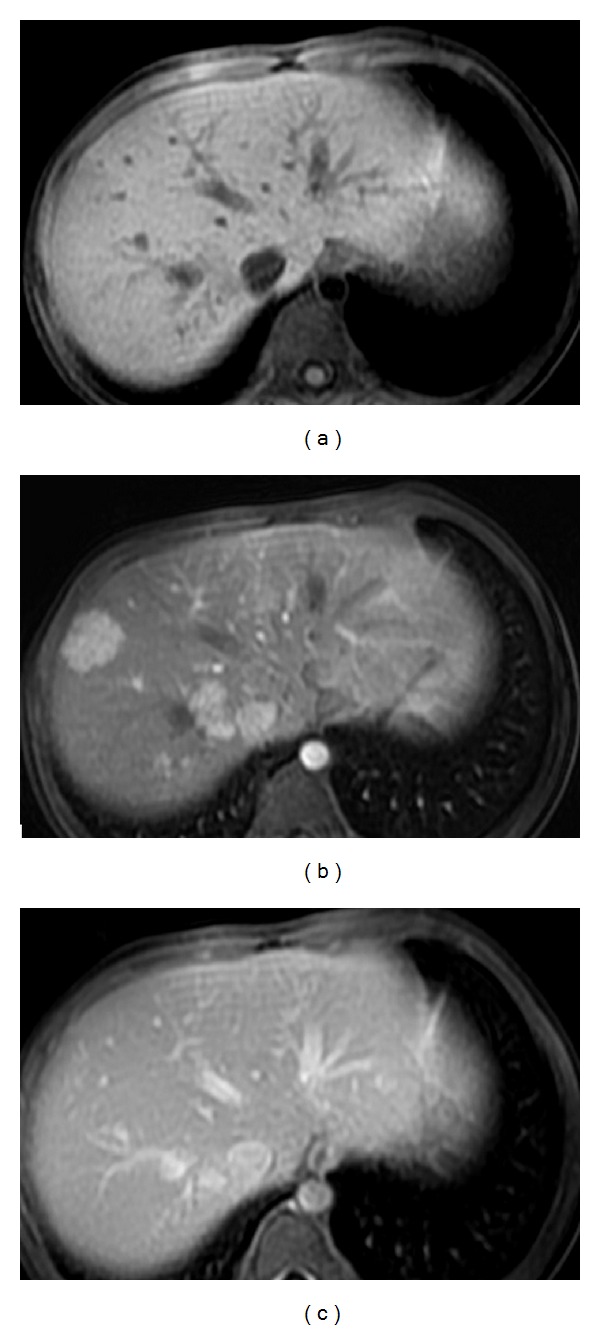
Nine-year-old girl with a history of metastatic right nephroblastoma treated with chemo- and radiotherapy. Liver MRI with T1-weighted images without (a) and with contrast injection at the arterial (b) and portal phases (c), performed six years after the end of treatment, displays multiple hepatic nodules, from 1 to 3 cm large, that enhance strongly after contrast injection at the arterial phase (b) and have almost the same signal as the surrounding liver before contrast injection (a) and at the portal phase (c). Simple clinical and imaging followup was performed (courtesy of Dr. H. Brisse, Institut Curie Paris, France).

**Figure 2:**
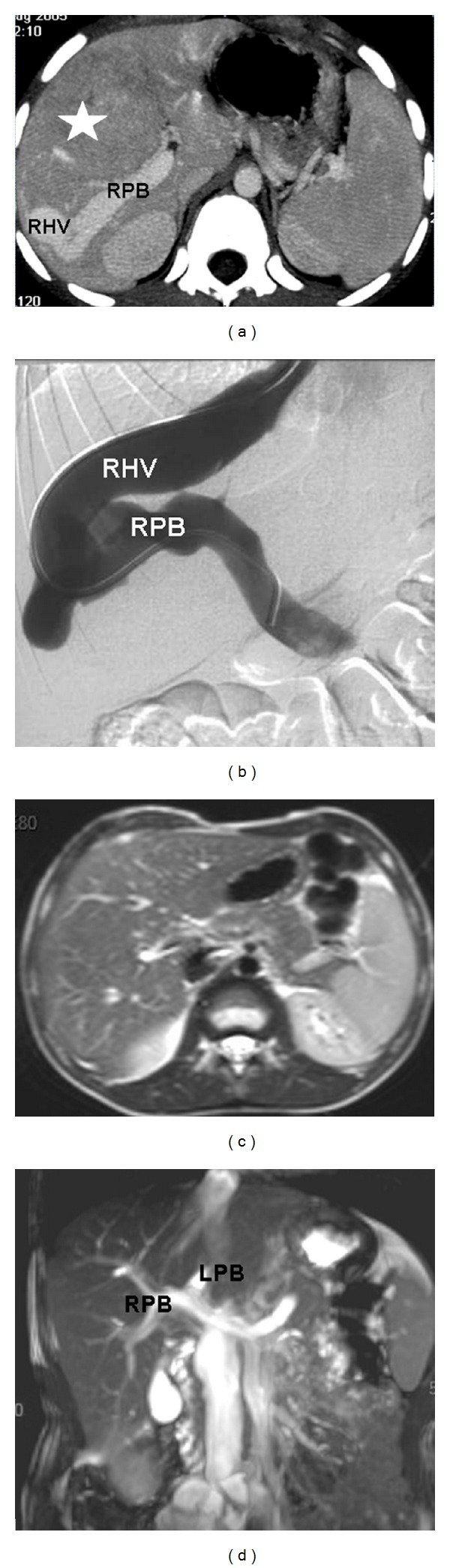
Six-year-old boy with a congenital portohepatic shunt complicated by a biopsy-proven FNH measuring 7 cm diameter (white star). (a) Contrast-enhanced CT scan at diagnosis shows an abnormal and large communication between the right portal branch (RPB) and the right hepatic vein (RHV). (b) Phlebography with opacification of the shunt between the RPB and the RHV. Closure of the shunt was performed by interventional radiology. (c) MRI performed seven years later shows the disappearance of FNH on the T2-weighted images. No enhancement was present at the arterial phase after gadolinium injection (not shown). (d) Note the normal aspect of the portal bifurcation (RPB and left portal branch (LPB)) on coronal MIP reconstruction of the T2-balanced sequence.

First known as an incidental lesion, FNH can also be associated with predisposing factors such as chemotherapy and radiation therapy in children treated for malignancy, and portal deprivation in case of congenital or surgical portosystemic shunts (CPSSs, SPSSs) (Figures 1 and 2). 

In the group of children with no predisposing factors, the incidence is estimated to be 0.5%. There is a female predominance as in adulthood. Mean age at diagnosis is between eight and 11 years [11].

In the population of long-term survivors of pediatric malignancy, the incidence of FNH is higher than in the general population and has been estimated to be 5%. This represents about one-third of children with FNH, but the number of cases reported is increasing as survivorship has significantly improved in the past decades. There is a male predominance, and mean age at diagnosis is older, between 10 and 16 years. Most patients have a history of malignancy or hematologic disorder requiring stem cell or bone marrow transplant (BMT). High doses of alkylating agents (busulfan and/or melphalan) that are very hepatotoxic and incriminated in hepatic venoocclusive disease and radiotherapy have been reported to be a risk factor for FNH. The mean time to develop FNH after treatment has been estimated to be between four and 12 years (from two to 27 years). This delay was shorter in children who had undergone high-dose chemotherapy along with BMT (7 years) (range 3–10 years). This delay was around 12 years (range 2–20 years) for patients who had not received this type of chemotherapy [9, 13, 19–21].

FNH has also been reported in children with congenital or surgical portosystemic shunt (CPSS and SPSS) and is probably secondary to complete or partial diversion of portal blood through the shunt, which leads to impaired portal blood supply with hyperarterialization of whole or part of the liver parenchyma [22, 23].

### 2.3. Clinical Presentation

Symptomatic FNH are more frequent in children than in adults and are found in about one-third of the patients. Two-thirds of the patients with tumors larger than 7 cm are symptomatic [11]. The most frequent symptom is abdominal pain. More rarely, weight loss and weakness can be encountered, mostly in very large tumors, and these symptoms can give concern for malignancy.

Patients who have a history of malignancy are more likely to have small asymptomatic lesions discovered on routine surveillance (Figure 1) [13, 21]. FNH associated with CPSS can be discovered either during routine surveillance of the vascular malformation or may be fortuitously discovered and then reveal the vascular malformation [22].

### 2.4. Imaging Features

FNH appears on US as a well-delimitated, lobulated mass that is iso- or slightly hypo- or hyperechoic compared to the surrounding liver. On Doppler examination, it is usually fed by a large artery with a stellate structure of its branches within the tumor. Typical radiologic findings on imaging techniques using contrast enhancement include a solitary, homogeneous, and slightly hypoattenuating mass compared to the surrounding liver on unenhanced CT with rapid homogeneous contrast enhancement at the arterial phase (except for the central scar). On venous phase, the mass becomes isodense as compared to the surrounding liver, while the central scar might be enhanced on the late phase. On MRI, FNH is usually hypo- or isointense to the surrounding liver on T1-weighted images and iso- to slightly hyperintense on T2-weighted images. Enhancement after gadolinium injection is similar to that observed on a CT scan [20, 24]. There is no calcification. In adults, due to the high specificity of CT and MRI in diagnosing FNH, there is usually no indication for biopsy in the presence of typical radiological features. In children, there is no study validating these criteria, but in our experience, when typical features of FNH are present, biopsy is not mandatory to confirm diagnosis. 

The diagnosis of FNH in children can be challenging as atypical lesions occur in about two-thirds of cases, and multiple lesions are more common in children than in adults [25]. Imaging features will depend on the context. In patients with no predisposing factor, FNH are frequently larger in children than in adults (64% of tumors >5 cm versus 20% in adults [9–11, 15, 26–29]. In most cases, arterial strong enhancement of the lesions on contrast-enhanced CT or MRI is present. It can be absent if there is a complete portal diversion secondary to CPSS or SPSS, as the surrounding liver may also be fed only by the hepatic artery. A central scar is very rare in the case of small or multiple FNH. It is better seen on MRI when present [11]. Fibrolamellar hepatocarcinoma is an important differential diagnosis as it frequently presents with an area of scarring. Patterns that are in favor of fibrolamellar hepatocarcinoma are lobulated margins of the tumor, the presence of calcifications, the large size of the central scar, the tumor heterogeneity before injection and at the arterial phase, and the presence of lymphadenopathies and/or the presence of metastases [30]. 

In the group of children with a history of chemo- and/or radiotherapy, a major concern is to differentiate benign and malignant processes. Strong enhancement at the arterial or early portal phase is important to differentiate FNH or “FNH-like” lesions from metastases that usually remain hypointense on arterial or early portal phase when compared to the surrounding liver [21]. Even if not presenting the typical diagnostic criteria for FNH, multiple liver lesions strongly enhancing at the arterial phase after injection in a long-term cancer survivor are highly suggestive of the diagnosis, and conservative treatment with imaging surveillance should be recommended (Figure 1) [11, 13, 21, 31].

### 2.5. Natural History and Management

**Figure 3:**
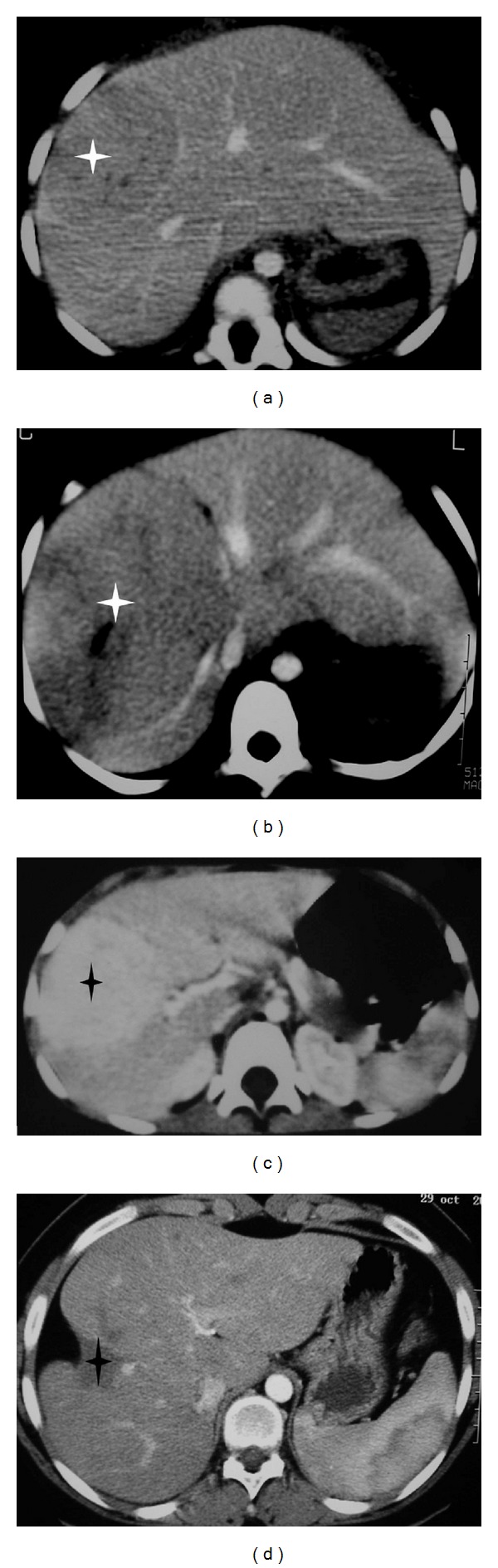
Spontaneous evolution of fortuitously discovered FNH in two children. (a) and (b): three-year-old girl with sickle-cell disease. CT scan after contrast injection at the portal phase at diagnosis (a). And three years later (b) shows growth of the tumor from 5 to 12 cm diameter. (c) and (d): eight-year-old girl. CT scan after contrast injection at diagnosis (c) and 12 years later (d) shows the spontaneous disappearance of the 5 cm diameter tumor with capsular retraction.

In the literature, few patients had conservative management of FNH. The natural history of these tumors is poorly known in children. All kinds of spontaneous evolution have been described, from spontaneous involution to growth (Figure 3) [11].

In the literature, 78% of the patients with no history of malignancy or hemopathy had a surgical resection. Most masses were large [11, 12].

In our practice, when the imaging features are typical on MR and/or CT, the patient is asymptomatic and there is no associated vascular disorder, biopsy is not performed, and conservative management is proposed with a prolonged followup by ultrasound to show if there is an increase in size.

When the aspect is not typical, biopsies of the mass and the nontumoral liver are performed to assess the diagnosis of the tumor and search for associated abnormalities of the liver that could be part of an unknown predisposing factor for a tumor.

Surgery is sometimes performed in the case of very large lesions, symptoms, and/or impairment of physical activities. Recurrence of FHN after surgery has been reported in one case in the literature, and we have a personal case, not published [32].

FNH secondary to CPSS requires special management as the closure of the shunt with restoration of intrahepatic portal flow may lead to shrinkage of the tumor, as shown in previous cases (Figure 2). That is why, whatever are the size of the tumor, the number of lesions, and their location, closure of the shunt should be performed when possible. We usually perform a biopsy of the tumor and the nontumoral liver to confirm the diagnosis of benign liver proliferation and exclude a hepatoportal sclerosis that would contraindicate the closure of the shunt [22].

In the case of SPSS, closure of the shunt should be discussed in regard to the patient's history. Restoration of intrahepatic portal flow by making a mesenterico-rex bypass should be performed when possible, mostly in the case of portal obstruction with cavernomatous transformation [23].

## 3. Hepatocellular Adenoma

### 3.1. Histopathological Definition

Hepatocellular adenomas (HCAs) are extremely rare during childhood. They are benign liver tumors that represent a heterogeneous group in which histopathological features may vary according to the etiological background. Classically, HCAs are soft, well-demarcated tumors with little or no fibrous capsule. They are composed of liver cell plate mildly thickened or irregular. They are supplied by thin-walled arteries without other portal tract elements (connective tissue, bile ducts, or ductular reaction).

In adults, genomic and molecular studies together with the analysis of genotype/phenotype correlations have led to the recognition of four major HCA subgroups: HNF1-*α*-inactivated HCA, *β*-catenin-activated HCA, and two forms of HCA without mutation of HNF1-*α* or *β*-catenin presenting either with or without inflammation. These different subtypes display variable clinical behavior, imaging findings, and natural history that have recently been well described [33–36]. To our knowledge, the only study concerning the profile of HCA genotype-phenotype in children concerns glycogen storage disease type I (GSD) and showed a high frequency of *β*-catenin mutations and lack of HNF 1*α* inactivation [37]. 

HCA formation is complex and varies according to the etiological background. Natural history and management vary with the context.

### 3.2. Epidemiology and Predisposing Factors in Children

According to the rare published pediatric series, HCA occurs in 0 to 21% of pediatric benign liver tumors. Differences in the frequency between series are probably related to the differences in patients' recruitments. The largest pediatric series reported 22 HCA in a 12-year period [12, 38–41]. 

Most HCAs are diagnosed during the teenage years (the mean age at diagnosis is around 14 years). HCAs reported before the age of one year are exceptional, the youngest patient being three weeks old in a context of multiple congenital anomalies [42].

Sex ratio varies with series, but the female predominance observed in adults is not the rule in children, and male predominance is observed in several series [38]. 

**Figure 4:**
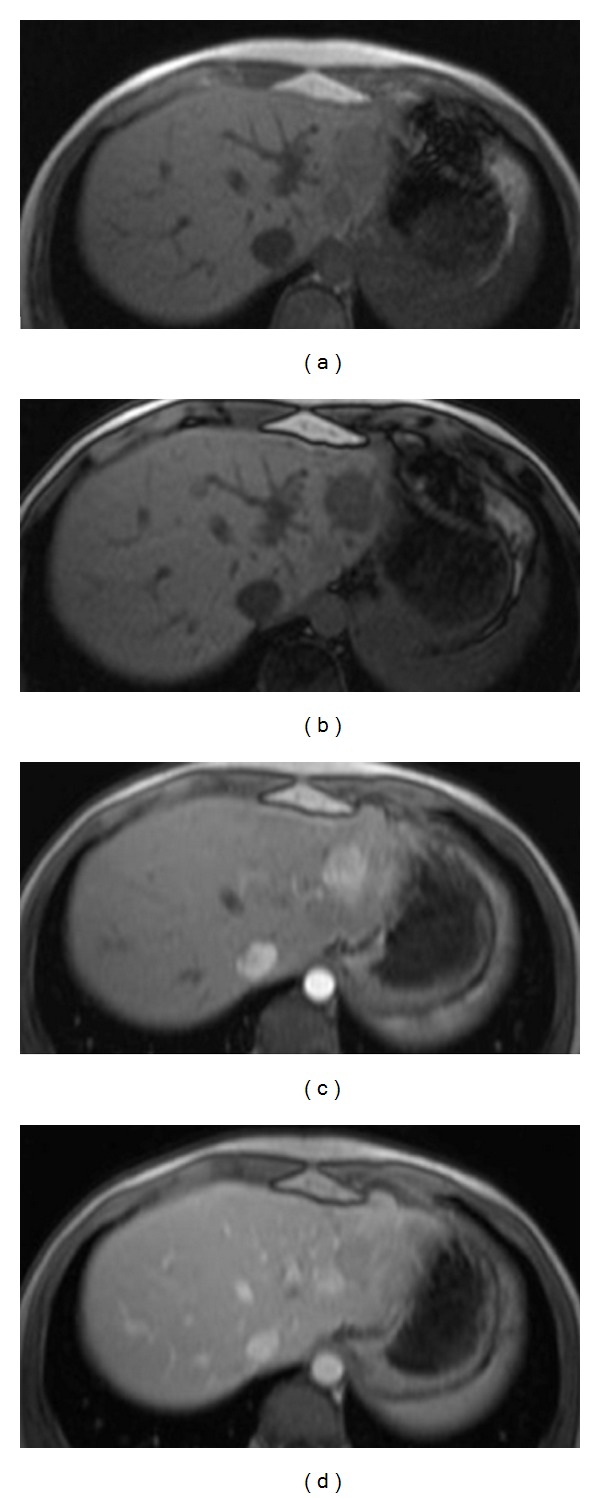
Adenomatosis related to HNF1-*α* germline mutation aspect on MRI in a 14-year-old girl: (a) and (b): T1 WI with chemical shift shows two lesions in the left lobe of the liver with drop of the signal of the largest lesion on the out-phase sequence that reveals the presence of fat in the tumor. (c) and (d): T1 WI after contrast injection shows early arterial enhancement of the largest lesion with washout in the late portal phase.

**Figure 5:**
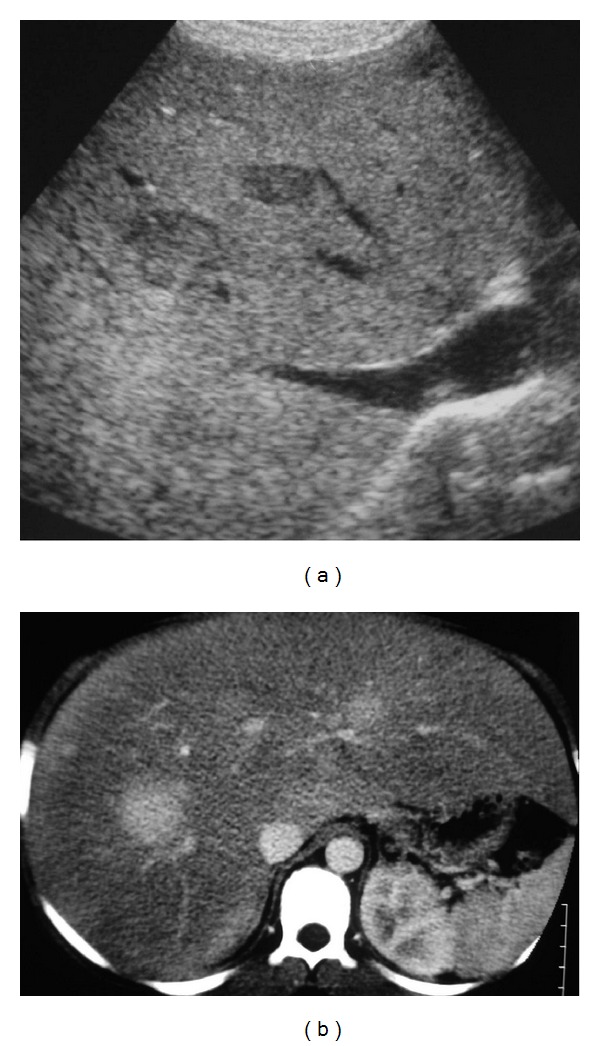
Fourteen-year-old boy with glycogen storage disease type I and multiple HCA measuring from 1 to 3 cm diameter. (a) US shows an enlarged hyperechoic liver (steatotic) with well-delimited hypoechoic nodules. (b) CT performed at the arterial phase after contrast injection shows enlarged steatotic liver with multiple nodules that are strongly enhanced.

**Figure 6:**
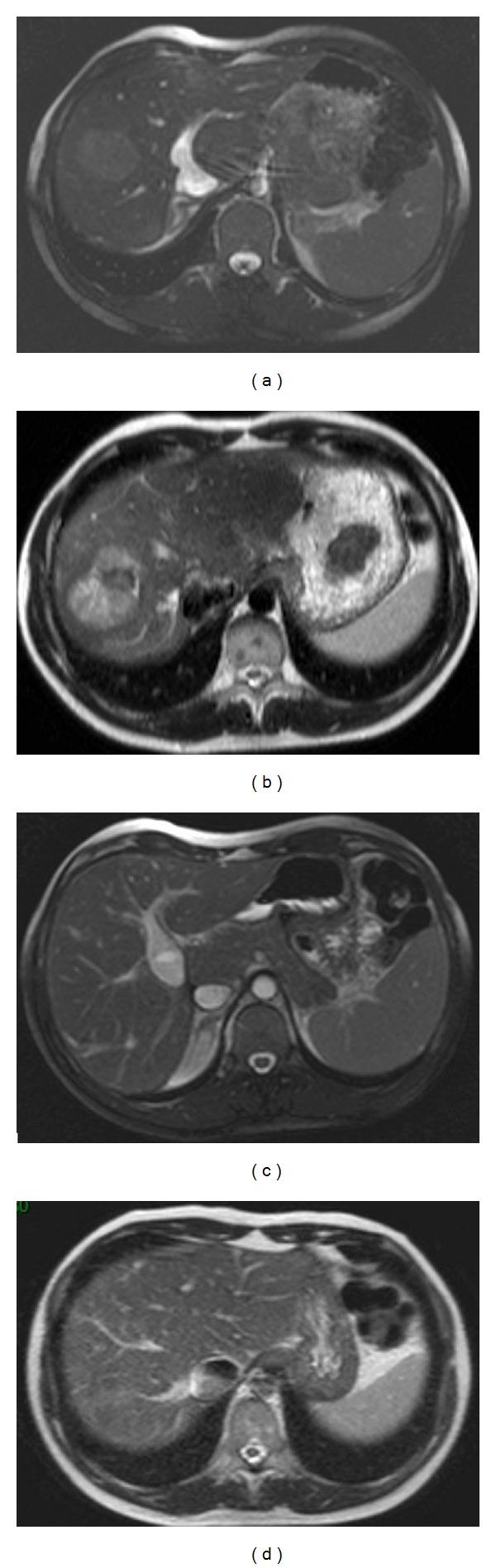
Adenoma associated with CPSS. Aspect on MRI at diagnosis and 10 months after surgical closure of the shunt: (a) T2 WI-balanced sequence shows the CPSS consisting of a patent ductus venosus. Note that the tumor is not easily visible in this sequence. (b) The 4 cm diameter HCA lies in segment 8, and it is better seen on T2 WI and appears heterogeneous and mainly hyperintense compared to the surrounding liver. MRI performed 10 months after surgical closure of the shunt (c) shows the complete disappearance of the tumor (d).

During childhood, HCA can be sporadic but is more frequently associated with predisposing factors such as GSD type I and III, anabolic androgenic steroid treatments with or without Fanconi anemia, congenital or surgical portosystemic shunt (CPSS, CPSS), germline mutation of HNF1-*α* gene, and familial adenomatosis polyposis (Figures 4, 5, and 6) [22, 23]. Hurler syndrome, Turcot syndrome, Lynch syndrome, immunodeficiency syndrome, tyrosinemia, and galactosemia have also been reported, and we have the personal unpublished experience of teenage girls with multiple HCA associated with Glanzmann's thrombasthenia treated by progestative therapy [43]. 

### 3.3. Imaging Features

Making the diagnosis of HCA by imaging can be challenging. In adults, major improvements in knowledge of HCA have been gained during recent years. Correlations of imaging with the genotype/phenotype classification proposed by the “Bordeaux” experience have made it possible to distinguish specific imaging patterns in relation with the two major subtypes, inflammatory HCA and HNF 1*α*-mutated HCA that account for 80% of all cases in adult series [34, 35, 44]. Very little data on the imaging features of HCA are available for children.

Ultrasonography is usually the first exam performed in children for the evaluation of abdominal disorders and the first screening tool during followup in predisposed children, but it can miss small nodules isoechoic to the liver, especially in a steatotic liver. HCAs are typically heterogeneous and are well-delimited solid masses with vessels within the mass (Figure 5). 

Magnetic resonance imaging (MRI) is the best technique to depict lesions. HCAs patterns on MRI will depend on the amount of fat, hemorrhage, and necrosis within the mass. HCA are frequently heterogeneous on T1- and T2-weighted images (WIs) but with a high signal on T2 WI. Fat component in HCA is well demonstrated with chemical shift sequences as in “in-phase and out-of-phase” T1 sequence. It can also be shown by sequences with fat saturations that are less sensitive. When present, it can help diagnosis. Early enhancement after contrast injection is observed in most cases. An enhanced pseudocapsule can be visible on delayed acquisition. Washout should not be too early (Figure 4). Diffusion sequences may help in the detection of small lesions. CT can also display fat within the tumor, heterogeneous content and early arterial enhancement [45]. In the presence of CPSS or SPSS, both HCA and nontumoral liver will lack portal vascularization, causing the absence of the early arterial enhancement classically observed in HCA, as both the nodule and the surrounding liver only have arterial supplies. 

When sporadic, HCA are frequently large solitary masses. In predisposed children, multiple HCA are more frequently observed. The term “adenomatosis” was first used in adult literature, and its definition excluded patients with GSD and steroid treatment [46]. This term has also been used for adenomas related to HNF1-*α* mutation (Figure 4).

Except for children with GSD, there are no published recommendations about screening protocols for HCA in patients presenting predisposing factors. At least annual ultrasonography should be performed, but MRI with its better sensitivity for the detection of liver tumors mostly in older children could be part of the systematic screening using T1-WI with fat suppression or chemical-shift sequences and T2-WI, diffusion sequences, and dynamic gadolinium injection when a lesion is detected on initial sequences.

An important concern is the detection of malignant transformation. This may be challenging and only possible with histology. Increasing size of the tumor and modifications of its aspect can be signs of malignant transformation. The kinetics of enhancement after contrast injection are important, and early washout after early enhancement at the arterial phase is suggestive of HCC transformation.

In most cases, histologic assessment of the tumor is necessary to adapt the management. In sporadic cases with no known predisposing factor, it is worthwhile to perform a biopsy on the nontumoral liver in order to depict an unknown underlying liver disease. 

### 3.4. Natural History and Predisposing Factors

In sporadic cases or when no predisposing factor is known, HCA can be found incidentally during imaging for unrelated pathology, but most often patients present with abdominal pain or palpable mass to the right upper quadrant or the epigastric region.

The two major concerns with HCA are hemorrhage and malignant transformation into HCC.

Hemorrhage is one of the most important complications of HCA. The maximum risks of bleeding and rupture have been estimated in 27.2 and 17.5 percent of patients respectively in a systematic review with the youngest patient being 14 years old [47]. Two types of hemorrhagic changes can take place in HCA: internal hemorrhage usually mixed with necrotic changes (usually in tumors larger than 4 cm) and spontaneous rupture with possible subcapsular hematoma and/or hemoperitoneum. Severe abdominal pain with possible hemodynamic disorders or even collapse can occur during intraperitoneal or intratumoral hemorrhage of HCA. Fatal issues have been reported in young patients with familial adenomatosis related to HNF1-*α* mutation and in FA. Hemorrhage has been reported in FA even after discontinuation of androgen therapy [48, 49].

*Malignant transformation* of HCA into HCC is rare, even in adults, and remains controversial in the literature [50]. Among the 50 cases (4.2%) of malignant transformation reported in a systematic review of 1635 HA (glycogenosis and adenomatosis were excluded), there were no pediatric cases [51]. In children, exceptional malignant transformations of HCA have been reported, mainly associated with GSD, FA with steroid therapy, and CPSS [22, 33, 51–55]. HCA associated with GSD type I displays high frequency of *β*-catenin mutation that could explain the high frequency of malignant transformation [37]. In adults, risk groups for malignant transformation of HCA are male gender, tumors larger than 5 cm, and *β*-catenin-activated HCA, and even though no data are yet published in the literature, these criteria should be taken into account when managing children. [51–53, 56].

In children with predisposing factors, the natural history and management of HCA will depend on the underlying pathology. 

*Concerning HCA complicating GSD type I and III,* most series or cases are associated with type I. In a series of 43 patients with GSD, about 52% of patients with type I and 25% of patients with type III glycogen storage disease had HCA [57]. Natural history and pathophysiological conditions remain poorly understood. HCA develop predominantly during and after puberty, between the ages of 10 and 20 years, and the incidence of new HCA appears to decrease after 20 years of age. The youngest patient presenting with HCA was 3.6 years old [58]. The male to female ratio is 1/1. The increased incidence of HCA development during adolescence may be related to suboptimal metabolic control during this period and/or to pubertal hormone secretion. Metabolic control seems to be an important factor for HCA development. Considerable alteration of lipid metabolism is a feature encountered in GSD associated with HCA formation. According to some authors, GSD type I displays a very high level of de novo fatty acid synthesis, which is known to play a role in tumorigenesis [58, 59]. 

Malignant transformation leading to HCC has been reported in adult patients and is probably related to the high frequency of *β*-catenin mutation [37, 52, 54, 55]. Recommendations for screening HCA by the “European Study of Glycogen Storage Disease Type I b” include an annual abdominal ultrasonography from birth to 10 years old, then every six months after 10 years of age. 

On liver ultrasound, HCAs are usually well-delimited round nodules hypoechoic compared to steatosis surrounding the liver parenchyma. If liver HCA is detected, ultrasonography should be performed every three months, associated with dosage of serum *α*-foetoprotein and carcinoembryonic antigen. CT or MRI with contrast injection will be performed on demand if the nodules increase in size, or if features suggestive of malignant transformation appear [60]. However, the difficulty in detecting HCA in bright liver with attenuation of US beam makes MRI more reliable for screening in some patients.

Management of HCA includes metabolic control as, when metabolic control is achieved, regression of HCA has been reported [61]. Surgery will depend on the presentation, ranging from tumorectomy to liver transplantation.

*In children with Fanconi Anemia (FA) and androgen therapy with or without FA,* liver tumors can occur and concern about 3% of patients [62]. Most are HCAs, but hepatocellular carcinoma (HCC), focal nodular hyperplasia (FNH), hepatoblastoma have also been reported [63–65]. Treatment of FA is based on androgen therapy and BMT as in other forms of aplastic anemia. Liver tumors can occur in FA patients in the absence of androgen therapy but are mainly associated with it. FA patients usually start androgens when they are young, at a median age of 7.5 years. Median duration of treatment with androgen prior to HCA or HCC diagnosis is four years, and median age at diagnosis of HCA is 12 years [64]. Association of HCA and HCC has been reported in several patients, and there is more likely to be transition from HCA into HCC as suggested by the presence of foci of HCC within HCA [63]. The median age at diagnosis of HCC in FA is 13.4 years [64]. Several factors may play a role in the development of HCA in FA: (i) genetic disorders and chromosomal defects allow mutagenesis and liver cell proliferation, (ii) chronic iron overload, which is frequently encountered even in the absence of blood transfusion or hemochromatosis, probably has a carcinogenetic effect [66, 67], and (iii) androgen therapy presents hepatic oncogenic properties [63, 64]. Screening for HCC in this context is difficult because HCC may occur despite typical radiological patterns of HCA. The *α*-foetoprotein test is not reliable as this biomarker has been found to be increased in about 85% of FA patients [67]. Close followup by imaging is mandatory for early diagnosis of HCA. When HCA is diagnosed, discontinuation of androgen therapy should be discussed if bone marrow function permits, as tumors may regress if androgens are withdrawn. Regression of HCA has also been reported after BMT. Close followup to depict transformation into HCC is mandatory and should be prolonged as late development of liver tumors (up to 24 years) is possible. Hemorrhagic complications have also been reported even after discontinuation of androgen therapy [49].

*Heterozygous germline mutations of the hepatocyte nuclear factor-HNF1 alpha* are associated with liver adenomatosis and maturity onset diabetes of the young (MODY 3) [46, 68–71]. Expression of the phenotype is variable for diabetes and adenomatosis. Severe intraperitoneal hemorrhages related to complicated adenomas have been reported, with a fatal issue in a sixteen-year-old girl [69]. Cases of malignant transformation have also been reported [68]. Adenomatosis has been reported in teenage patients with the youngest being 14 years old [48]. Systematic screening should be performed in relatives of patients with liver adenomatosis and should start at the age of ten years. Ultrasonography is a good screening tool, but adenomas may be difficult to diagnose in some patients. MRI with contrast injection should be performed to increase the sensitivity of screening (Figure 4). A CT scan with contrast injection can also be proposed if MRI is not available. Adenomas are often steatotic. Serum *α*-foetoprotein levels should also be part of the screening. If liver adenomatosis is detected, survey and preventive surgical treatment should be discussed. Criteria that guide treatment include the number and size of the lesions, the presence of symptoms, and the surgical risk incurred by the patient. 

*Congenital or Surgical portosystemic shunts are associated with HCA,* as reported in several cases [22, 23, 72]. Partial or complete diversion of the portal flow through the shunt leads to an abnormal hepatic circulation that may cause hepatocytic proliferation with nodule formation [8]. Regression of adenomas has been observed after closure of CPSS and restoration of portal blood flow (Figure 6). 

### 3.5. Management

It is now well established in adults that complications depend not on the number of lesions but on the histologic subtype and size of the tumor. In adults, complete surgical resection is an effective option for HCA larger than 5 cm or in male or if HCA is associated with GSD. In our personal pediatric experience and in the pediatric literature, HCAs which are frequently larger than 5 cm are resected [38, 40]. Surgical resection should also be discussed in case of *β*-catenin mutation or uncertain diagnosis. Some authors propose resection of smaller HCA, between 3 and 5 cm, because of reported cases of malignant transformation [53, 73]. The surgical technique will depend on the context, location, and size of the tumor, and it may consist of a tumorectomy, atypical resection, or hepatectomy. Liver transplantation has been proposed by some authors in the case of multiple lesions [48, 74, 75]. In our experience and as suggested by Dokmak and coll. in adult patients, we propose a conservative treatment for multiple HCA or adenomatosis, involving resection of the largest tumors and close followup of the remaining small lesions with iterative resection if necessary. The place of percutaneous ablation using either radiofrequency, cryotherapy, or other techniques is still unclear [76].

Embolization can be performed in the case of hemorrhage.

With predisposing factors such as GSD, androgen steroid therapy, CPSS, or SPSS, regression of HCA has been observed with metabolic control, androgen withdrawn, and closure of the shunt, respectively.

## 4. Conclusion

Most FNH remain sporadic during childhood, but predisposing factors exist, as follows: long-term cancer survivor (with an increasing incidence) and portal deprivation in CPSS and SPSS. The aspect is atypical on imaging in two-thirds of cases. Biopsy of the tumor and the nontumoral liver is then required. Surgical resection will be discussed in the case of large tumors with or without symptoms. In the case of associated vascular disorder with portal deprivation, restoration of the portal flow will be discussed. This will be done either by closure of a congenital portosystemic shunt or by making a mesenterico-rex bypass in the cavernous transformation of the portal vein in the hope of seeing the involution of FNH.

HCA, which is very rare in children, is frequently associated with predisposing factors such as GSD type I and III, Fanconi anemia especially if androgen therapy is administered, CPSS, and SPSS. Adenomatosis has been reported in germline mutation of HNF1-*α*. Management will depend on the presence of a predisposing factor and may include metabolic control, androgen therapy withdrawn, or closure of the shunt when appropriate. Surgery is usually performed on large lesions. In the case of adenomatosis or multiple lesions, surgery will be adapted. Close followup is required in all cases.
